# Supplementary material for: Germination Promotes Flavonoid Accumulation of Finger Millet (Eleusine coracana L.): Response Surface Optimization and Investigation of Accumulation Mechanism
Source: Plants (Basel). 2024 Aug 8;13(16):2191. doi: 10.3390/plants13162191 (PMC11360649; doi:10.3390/plants13162191)
Supplement: Supplementary file 1 [file plants-13-02191-s001.zip › plants-3104360-supplementary.pdf]

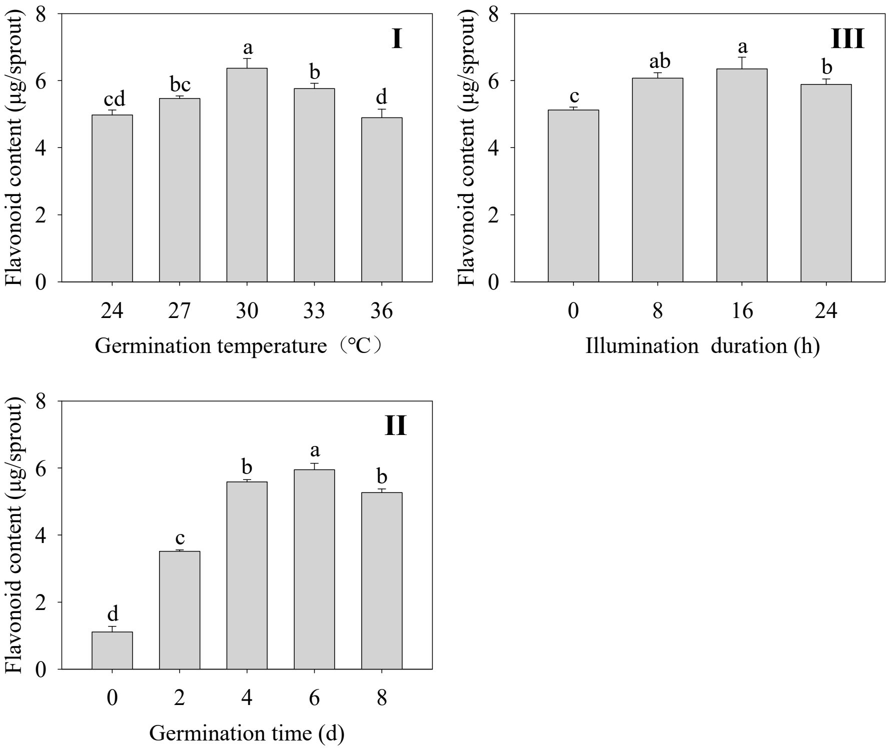


**Figure S1** Effects of Germination temperature (**I**), Germination time (**II**), and Illumination duration (**III**) on flavonoid content in finger millet sprouts. Each data point represents the average (average ± SD) of three independent biological replicates. Lowercase letters reflect significant differences ($p < 0.05$) in indicators between different germination time using Tukey's test.

**Table S1** Encoded and real levels for the germination process of finger millet sprout and the flavonoid content of each treatment group

| **Independent variables** | **Levels** | | |
|---|---|---|---|
| | **-1** | **0** | **1** |
| $X_1$: Germination time (d) | 4 | 5 | 6 |
| $X_2$: Germination temperature (°C) | 27 | 30 | 33 |
| $X_3$: Illumination duration (h) | 8 | 16 | 24 |

| **No** | **$X_1$: Germination time (d)** | **$X_2$: Germination temperature (°C)** | **$X_3$: Illumination duration (h)** | **Y: Flavonoid content (μg/sprout)** |
|---|---|---|---|---|
| 1 | 6 | 30 | 8 | 6.785 |
| 2 | 6 | 30 | 24 | 6.84252 |
| 3 | 5 | 30 | 16 | 6.91342 |
| 4 | 6 | 33 | 16 | 6.76 |
| 5 | 4 | 30 | 24 | 6.76689 |
| 6 | 4 | 30 | 8 | 6.4886 |
| 7 | 6 | 27 | 16 | 5.12495 |
| 8 | 5 | 33 | 8 | 6.377 |
| 9 | 5 | 30 | 16 | 6.95132 |
| 10 | 5 | 30 | 16 | 6.97778 |
| 11 | 5 | 33 | 24 | 6.583 |
| 12 | 4 | 27 | 16 | 5.3686 |
| 13 | 4 | 33 | 16 | 6.33123 |
| 14 | 5 | 30 | 16 | 6.91313 |
| 15 | 5 | 27 | 8 | 5.16347 |
| 16 | 5 | 30 | 16 | 6.96121 |
| 17 | 5 | 27 | 24 | 5.2695 |

**Table S2** The sequences of the primers utilized in the study

| Gene Name | Fordward Primer (5' - 3') | Reverse Primer (5' - 3') |
|---|---|---|
| *Actin* | CTCACGCTCAAGTACCCAATC | GGCAACACGAAGCTCATTGTA |
| *Ubiqutin* | CAAGACTGCGAAGGACTACAA | ACAGATTGATGACTGCCTCTTTA |
| *CAT* | ACCCGCCTTTACTACTTTTT | CATAGCCGAAAAGCATCCAT |
| *SOD* | CTCCTACGGCGACCTCTACCAGC | CTGAGGCTTGTCCTCCCTCCCTG |
| *POD* | CCAGGTGCTCTACTCCGACGACC | GAGGTTGGTCATGGCGGCGAC |
| *APX* | CACCTGTTCCTCGACTTTGC | TTACGTTGCAGCAGTTGAGG |
| *PAL* | CGTGCCGCTCTCCTACATTGC | CCTCTGCTGCGTTCACCTTGG |
| *C4H* | GACTTCCGCTTCCTGCCGTTC | CACGAGCTTGCCGACGATGAG |
| *4CL* | GACGACAAGGCGACCAAGGC | CTCCACGCTGCTGATGTTCTCG |
| *CHI* | GCCGCCGTGGAGAAGTTCAAG | ACCGACGAGTCCTTGGAGAACG |
| *CHR* | AGTCTCAAGATCGCATTGCTGGTG | AACTTGTGGTGAGGTGTGCTGTG |
| *CHS* | ATGCTGTTCTCCGTCCCGAATTTC | CTTATCTTCCTGGCGAGCACCTTC |
| *MYB* | AGGAGGAGGAAGATGCTGAAAGT | TTGAGGTGGTTGGATAGTGAGAG |
